# A Study of Functional Outcome of Proximal Tibia Fractures Treated With Minimally Invasive Percutaneous Plate Osteosynthesis Technique: A Prospective Study

**DOI:** 10.7759/cureus.104526

**Published:** 2026-03-02

**Authors:** Basanth Reddy A, Nagakumar J S., Gils Thampi, Navin Balaji R

**Affiliations:** 1 Orthopedics, Sri Devaraj Urs Medical College, Kolar, IND

**Keywords:** functional outcome, locking compression plate, minimally invasive plate osteosynthesis, proximal tibia fracture, rasmussen functional score, tibial plateau fracture

## Abstract

Background

Proximal tibial fractures are complex periarticular injuries that commonly result from high-energy trauma and are frequently associated with articular incongruity, instability, and long-term functional impairment. Restoration of joint congruity, along with preservation of the soft tissue envelope, is essential to achieve a satisfactory outcome. Minimally invasive percutaneous plate osteosynthesis (MIPPO) has gained popularity because it provides stable fixation while preserving fracture biology and minimizing complications related to extensive surgical exposure.

Materials and methods

A prospective observational study was conducted on 35 adult patients with closed proximal tibial fractures treated with locking compression plates using the MIPPO technique. Patients were evaluated clinically and radiologically at regular intervals for up to six months postoperatively. Functional outcomes were assessed using the modified Rasmussen functional score, and pain severity was measured using the visual analog scale (VAS). Radiological union, range of motion, and complications were also documented.

Results

The mean age of the patients was in the fourth decade, with a male predominance. Road traffic accidents were the most common mechanism of injury. Most fractures were classified as Schatzker type IV and V patterns. The average radiological union time was approximately 20 weeks. The mean VAS score progressively decreased, while the modified Rasmussen score significantly improved during follow-up. At the final evaluation, the majority of patients achieved excellent to good functional outcomes with a satisfactory range of motion in the knee. Complications were minimal and included isolated cases of superficial infection, stiffness, and malalignment.

Conclusion

MIPPO, utilizing a locking compression plate, offers reliable fixation for proximal tibial fractures while preserving vascularity and the fracture hematoma. This technique enables early mobilization, promotes predictable fracture union, and yields favorable functional outcomes with a low complication rate, making it an effective treatment modality for peri-articular proximal tibial injuries. This study has been reported in accordance with the STROBE guidelines for observational studies.

## Introduction

Proximal tibial fractures account for approximately 5-11% of all tibial fractures and often result from high-energy trauma, such as road traffic accidents or falls [1]. These injuries frequently involve the articular cartilage and associated ligamentous structures, which can lead to long-term functional disability if not properly treated [2]. The primary goals of management are to restore joint congruity, maintain limb alignment and knee stability, and promote early mobilization.

The lateral plateau is most frequently involved, while bicondylar fractures represent more severe patterns that require stable fixation and precise reduction [3]. Malalignment alters load distribution across the knee, predisposing it to post-traumatic osteoarthritis and stiffness [4]. Therefore, anatomical reduction and stable fixation are essential to restore knee biomechanics and function.

Traditional open reduction techniques require extensive soft tissue dissection and are associated with wound complications and infections due to periosteal stripping [5]. External fixation minimizes soft tissue damage but may lead to pin-tract infections and delayed joint mobilization [6]. Therefore, an ideal fixation method should provide stability while preserving soft tissues.

Biological fixation emphasizes preserving the periosteal blood supply and fracture hematoma to promote healing [7]. Minimally invasive percutaneous plate osteosynthesis (MIPPO) achieves indirect reduction and bridging fixation without disrupting fracture biology. Locking compression plates function as internal fixators, providing angular stability and maintaining alignment, particularly in metaphyseal bone [8,9].

The primary objective of this study was to evaluate the radiological union time and functional outcomes - assessed using the modified Rasmussen score and visual analog scale (VAS) - in patients with closed proximal tibial fractures treated with MIPPO over a six-month follow-up period. The secondary objectives were to assess complication rates, knee range of motion, and the maintenance of limb alignment following surgical management.

## Materials and methods

This prospective observational cohort study was conducted at a tertiary care teaching hospital over eighteen months, following institutional ethical approval (approval number SDUMC/KLR/IEC/304/2022-23) and obtaining written informed consent from all participants. Thirty-five skeletally mature patients with closed proximal tibial fractures were included. The inclusion criteria comprised skeletally mature patients presenting with closed proximal tibial fractures within one week of injury. Exclusion criteria were open fractures, pathological fractures, polytrauma requiring staged management, previous knee pathology, and associated neurovascular injuries. The mechanism of injury, associated injuries, comorbidities, and soft tissue condition were documented upon admission. Preoperative neurovascular status of the affected limb was assessed in all patients. Temporary immobilization with an above-knee slab and limb elevation were provided until surgery.

Patients presenting within one week of injury underwent clinical and radiographic evaluation using anteroposterior and lateral knee radiographs. Fractures were classified according to the Schatzker classification. A CT scan with three-dimensional reconstruction was performed for comminuted or depressed fractures to assess articular involvement and plan screw trajectory and plate positioning. Surgery was performed after the subsidence of soft tissue swelling and the appearance of the wrinkle sign.

Routine preoperative investigations and anesthetic clearance were obtained. A single preoperative intravenous dose of ceftriaxone (1 g) was administered within 30 minutes prior to the incision, followed by postoperative antibiotics for 48 hours according to the institutional protocol. All procedures were performed under spinal or combined spinal-epidural anesthesia, with the patient in the supine position on a radiolucent table. A tourniquet was applied when the soft tissue condition permitted. Closed reduction was achieved using manual traction, ligamentotaxis, and percutaneous clamps under fluoroscopic guidance to restore alignment. Depressed articular fragments, when present, were elevated using a bone tamp through a cortical window.

Through small proximal and distal incisions, a submuscular, extraperiosteal tunnel was created along the lateral proximal tibia. A precontoured proximal tibial locking compression plate was then inserted using a MIPPO technique. Proximal locking screws stabilized the articular fragment, while distal cortical or locking screws restored length and alignment. The plate length was selected to provide adequate working length, with at least three bicortical screws placed distal to the fracture. Screw density was kept low to preserve construct elasticity. Depressed fragments were supported with autologous cancellous bone grafts when necessary. Wounds were irrigated and closed in layers, followed by sterile dressing.

Postoperatively, ankle pumps and quadriceps isometric exercises were initiated on the first day after surgery, and knee mobilization began within 48 hours. Active-assisted range-of-motion and progressive quadriceps strengthening exercises continued under supervision. Patients remained non-weight bearing for six weeks, after which partial weight bearing was permitted based on radiological evidence of callus formation and was gradually advanced to full weight bearing once union was confirmed.

Patients were evaluated at one, three, and six months. Assessments included pain measurement using the VAS [10], knee range of motion, radiological union, complications, and functional evaluation using the Modified Rasmussen Score [4]. The modified Rasmussen functional score assesses pain, walking capacity, range of motion, stability, and the ability to return to work. The total score ranges from 0 to 30, with outcomes classified as excellent (27-30), good (20-26), fair (10-19), and poor (<10). Functional scoring was conducted by an independent orthopedic surgeon who was not involved in the initial procedure. Radiological union was defined as the presence of bridging callus across at least three cortices on anteroposterior and lateral radiographs, accompanied by the absence of pain at the fracture site during weight-bearing. The VAS described by Huskisson and the Rasmussen scoring system are public-domain clinical assessment tools and do not require permission for academic research use.

Consecutive eligible patients were included to minimize selection bias. Clinical and radiological assessments followed predefined criteria, and radiographs were independently evaluated by two orthopedic surgeons who were not involved in the procedure to reduce observer bias. Any disagreements were resolved by consensus.

Potential confounding variables, including age, sex, mechanism of injury, fracture type (Schatzker classification), soft tissue condition, associated injuries, comorbidities, and time to surgery, were recorded at baseline. Patients with open fractures, pathological fractures, or neurovascular injuries were excluded to minimize confounding. Functional and radiological outcomes were analyzed across follow-up intervals using within-subject comparisons to reduce inter-patient variability. As all patients underwent the same surgical technique and rehabilitation protocol, treatment-related confounding was minimized.

As this was a prospective observational cohort study conducted within a defined study period, all eligible patients presenting during the study duration were included. A formal sample size calculation was not performed.

Statistical analysis

Continuous variables were tested for normality and expressed as mean ± standard deviation or median (interquartile range), as appropriate, while categorical variables were presented as frequencies and percentages. Repeated-measures ANOVA with post hoc pairwise comparisons was used for longitudinal comparisons of functional and pain scores, and the chi-square test was applied for categorical variables. Statistical analysis was performed using IBM SPSS Statistics for Windows, Version 25.0 (IBM Corp., Armonk, NY, USA). A p-value of <0.05 was considered statistically significant.

This study was reported in accordance with the Strengthening the Reporting of Observational Studies in Epidemiology (STROBE) guidelines. Patient confidentiality was maintained in accordance with the Declaration of Helsinki.

## Results

All enrolled patients completed the six-month follow-up and were included in the final analysis; no patients were lost to follow-up. Thirty-five patients with proximal tibial fractures were included in the study. Baseline demographic and clinical characteristics are presented in Table 1. The mean age was 43.2 years, with a male predominance (74.3%). Road traffic accidents (68.6%) were the most common mechanism of injury. Most patients had closed fractures with mild soft tissue involvement, and the mean time to surgery was 4.6 days.

**Table 1:** Baseline demographic and clinical characteristics of patients (n = 35) Values are presented as mean ± standard deviation or number (%). Soft tissue injuries were graded according to the Tscherne classification for closed fractures. No comparative statistical test was applied as the table summarizes baseline characteristics.

Variable	Category	Value
Age	Mean age (years)	43.2 ± 12.6
Sex	Male	26 (74.3%)
	Female	9 (25.7%)
Side involved	Right	19 (54.3%)
	Left	16 (45.7%)
Mode of injury	Road traffic accident	24 (68.6%)
	Fall from height	8 (22.9%)
	Domestic fall	3 (8.5%)
Associated injuries	Present	6 (17.1%)
	Absent	29 (82.9%)
Comorbidity	Diabetes mellitus	5 (14.3%)
	Hypertension	4 (11.4%)
	None	26 (74.3%)
Time to surgery	Mean days	4.6 ± 1.8
Soft tissue injury (Tscherne classification)	Grade 0	12 (34.3%)
	Grade 1	16 (45.7%)
	Grade 2	7 (20.0%)

The study population predominantly consisted of middle-aged individuals, with 17 (48.6%) patients in the 31- to 50-year age group, followed by 10 (28.5%) patients aged 51-70 years and 8 (22.9%) patients aged 18-30 years. The distribution was not statistically significant (χ² = 1.74, p = 0.41) (Table 2).

**Table 2:** Age distribution (n = 35) Values are expressed as number (%). The chi-square test was used to compare age group distribution.

Age group (years)	Number	Percentage (%)	p-value	Test statistic (χ²)
18–30	8	22.9	0.18	1.74
31–50	17	48.6	0.004	1.74
51–70	10	28.5	0.21	1.74

**Figure 1:**
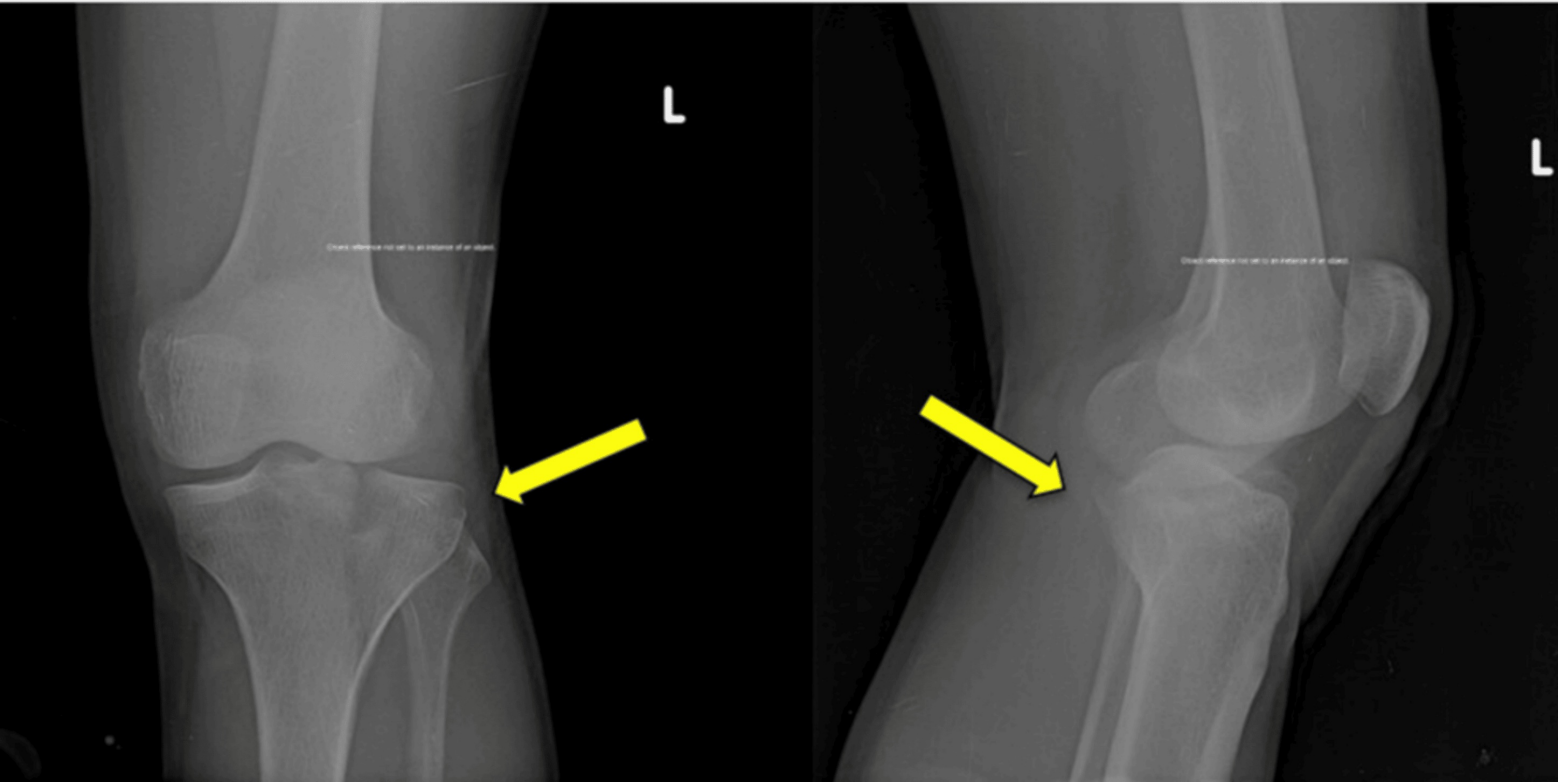
Preoperative anteroposterior and lateral radiographs demonstrating a proximal tibial fracture.

**Figure 2:**
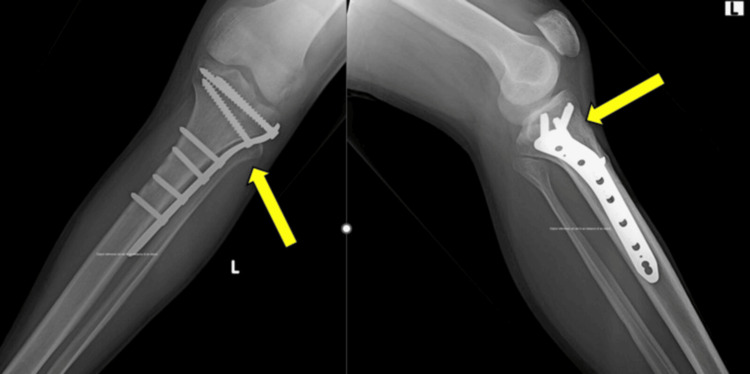
Immediate postoperative radiographs showing fixation with proximal tibial locking compression plate using minimally invasive percutaneous plate osteosynthesis technique.

Schatzker type IV fractures were the most frequent, observed in 11 patients (31.4%), followed by type V in 7 (20.0%) patients and type II in 6 (17.1%) patients. The predominance of higher-grade fractures was statistically significant (χ² = 12.63, p = 0.027), indicating a referral pattern for complex periarticular injuries (Table 3). A representative preoperative anteroposterior and lateral radiograph demonstrating a proximal tibial fracture is shown in Figure 1. Immediate postoperative anteroposterior and lateral radiographs demonstrating fixation with a proximal tibial locking compression plate are shown in Figure 2.

**Table 3:** Schatzker fracture classification (n = 35) Values are expressed as number (%). The chi-square test was applied to assess the distribution of fracture types.

Schatzker type	Number	Percentage (%)	p-Value	Test statistic (χ²)
I	3	8.6	0.12	12.63
II	6	17.1	0.29	12.63
III	4	11.4	0.18	12.63
IV	11	31.4	0.002	12.63
V	7	20	0.07	12.63
VI	4	11.4	0.18	12.63

The mean VAS pain score progressively decreased from 6.2 at one month to 3.4 at three months and 1.5 at six months. This reduction was statistically significant (F = 148.2, p < 0.001), indicating effective stabilization and postoperative symptomatic relief (Table 4).

**Table 4:** Pain assessment using VAS score (n = 35) Values represent mean scores at each follow-up. Repeated-measures analysis of variance (ANOVA) was used for longitudinal comparison of pain scores. VAS, visual analog scale

Follow-up	Mean VAS	p-Value	Test statistic (F)
1 month	6.2	<0.001	148.2
3 months	3.4	<0.001	148.2
6 months	1.5	<0.001	148.2

The mean modified Rasmussen functional score improved from 16.8 at one month to 23.4 at three months and 27.6 at six months. This improvement was statistically significant (F = 132.6, p < 0.001), indicating progressive functional recovery associated with fracture healing (Table 5).

**Table 5:** Functional assessment over follow-up using the modified Rasmussen score (n = 35) Values represent mean functional scores at each follow-up. Repeated-measures analysis of variance (ANOVA) was used for longitudinal comparison of functional outcomes.

Follow-up	Mean score	p-Value	Test statistic (F)
1 month	16.8	<0.001	132.6
3 months	23.4	<0.001	132.6
6 months	27.6	<0.001	132.6

Excellent outcomes were achieved in 18 (51.4%) patients and good outcomes in 12 (34.3%) patients, resulting in an overall satisfactory rate of 85.7%. Fair results were observed in four (11.4%) patients and poor outcomes in one (2.9%) patients. The distribution demonstrated statistical significance favoring satisfactory outcomes (χ² = 16.94, p = 0.001) (Table 6).

**Table 6:** Final functional outcome according to the modified Rasmussen grading system (n = 35) Values are expressed as number (%). The chi-square test was used to analyze the distribution of final functional outcomes based on the Modified Rasmussen scoring system.

Outcome	Number	Percentage (%)	p-Value	Test statistic (χ²)
Excellent	18	51.4	0.001	16.94
Good	12	34.3	0.02	16.94
Fair	4	11.4	0.11	16.94
Poor	1	2.9	0.36	16.94

**Figure 3:**
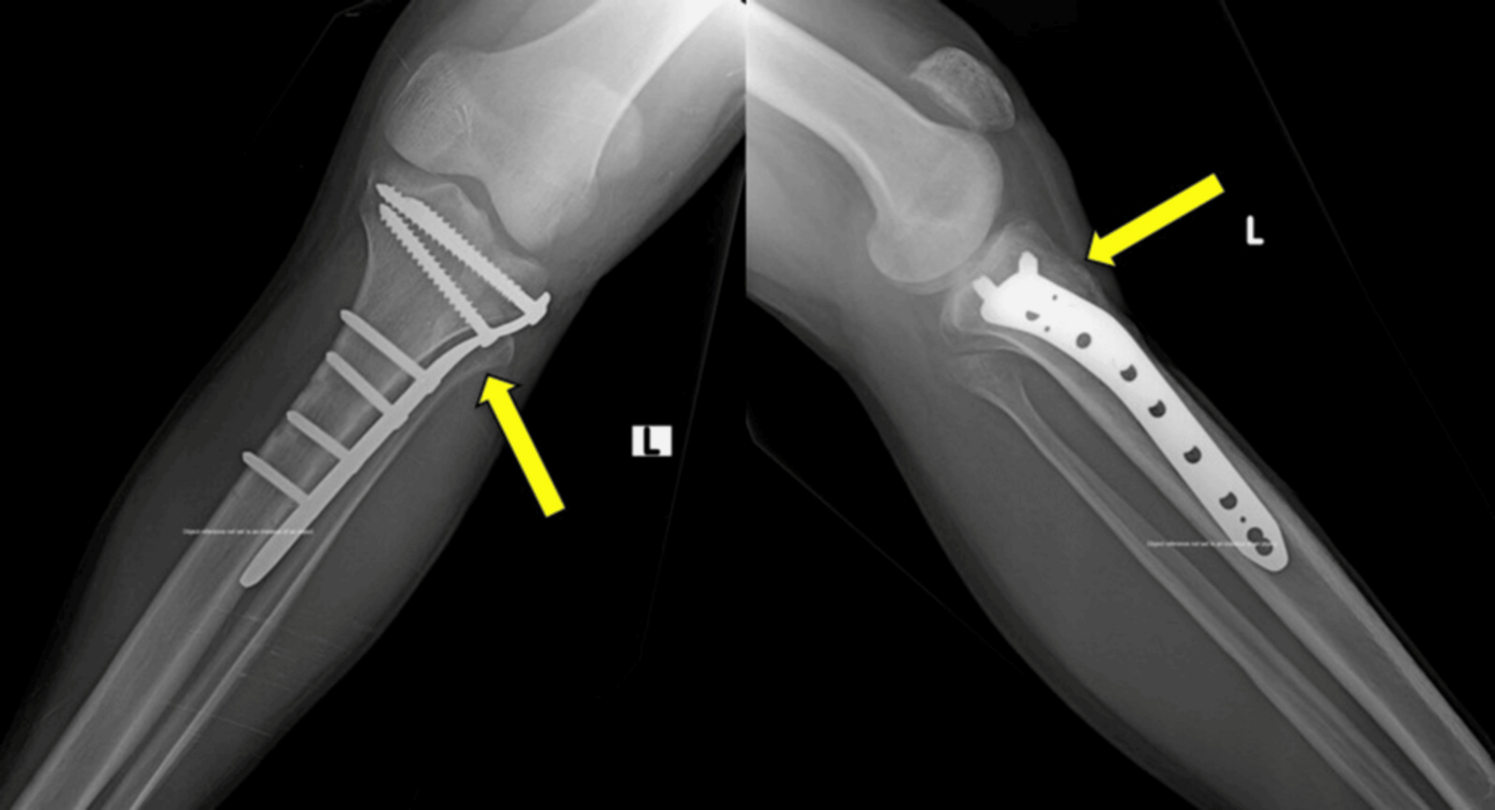
Follow-up radiographs at six months demonstrating fracture union with maintained alignment.

The mean radiological union time observed in the study was approximately 20 weeks (95% CI, 18.9-21.1 weeks). Follow-up anteroposterior and lateral radiographs at six months demonstrated fracture union with maintained alignment (Figure 3). A few complications were noted during follow-up, including isolated cases of superficial infection, knee stiffness, and mild malalignment. All were managed conservatively without the need for implant removal.

## Discussion

Management of proximal tibial fractures requires the restoration of articular congruity, mechanical axis alignment, and knee stability [11]. Failure to achieve anatomical reduction results in abnormal load transmission across the knee joint, accelerating degenerative changes. Historically, open plating achieved accurate reduction but compromised soft tissue integrity, with reported infection rates approaching 20% due to periosteal stripping and vascular compromise [12].

MIPPO adheres to the principles of biological fixation by preserving the periosteal blood supply and fracture hematoma, thereby promoting faster and more predictable healing [13]. Locking plates function as internal fixators and are especially beneficial in metaphyseal bone, where screw purchase is limited and conventional plating may fail [14]. By acting as a bridging construct, they provide angular stability while maintaining alignment without excessive exposure of the fracture site.

In the present study, the average union time was comparable to the findings reported by Cole et al. [15] and Egol et al. [16]. Functional outcomes were also similar to those reported by Oh et al. [17], who observed excellent-to-good results in the majority of patients treated with minimally invasive fixation. The low infection rate noted in our series correlates with minimal surgical dissection and reduced soft tissue handling compared to the open reduction techniques described by Barei et al. [11].

Pain reduction and improvement in range of motion were progressively observed during follow-up, owing to stable fixation that permitted early rehabilitation [18]. Maintenance of alignment was achieved as the bridging plate neutralized deforming forces while preserving fracture biology. Preservation of vascularity promotes callus formation and reduces the risk of delayed union, which explains the consistent improvement in functional scores over time.

Early mobilization following stable fixation reduces peri-articular fibrosis and prevents stiffness, a common complication in tibial plateau fractures. Minimizing surgical trauma also decreases postoperative swelling and inflammatory response, enhancing patient compliance with physiotherapy and facilitating a quicker return to daily activities. The low complication rate observed in this study supports the effectiveness of minimally invasive fixation compared to traditional open plating.

Previous literature also emphasizes that fracture classification helps predict prognosis and guides fixation strategy. The Schatzker classification remains clinically relevant for determining fracture severity and surgical planning [19]. Furthermore, modern management protocols recommend stable fixation combined with early mobilization to optimize functional recovery and prevent long-term disability in tibial plateau fractures [20].

The findings are generalizable to similar tertiary trauma care settings that manage peri-articular tibial fractures using minimally invasive fixation techniques.

Limitations

This study has several limitations. The relatively small sample size reduces the statistical power for subgroup analyses and may limit the generalizability of the findings. The six-month follow-up period captures only early outcomes and may not reflect long-term degenerative changes, such as post-traumatic osteoarthritis or residual instability. The lack of a control group prevents causal inferences about the superiority of the MIPPO technique. Furthermore, as this was a single-center study, the external validity of the results may be limited.

## Conclusions

MIPPO using a locking compression plate is an effective treatment option for closed proximal tibial fractures. In this prospective observational cohort study, the technique was associated with predictable radiological union, significant improvements in pain and functional scores, satisfactory short-term outcomes, and a low complication rate. Early mobilization and preservation of soft tissues likely contributed to the favorable recovery. However, given the absence of a control group, the relatively small sample size, and the short follow-up duration, these findings reflect only short-term outcomes and should be interpreted within the limitations of the study design. Further comparative studies with larger cohorts and longer follow-up periods are warranted to validate these results.
